# Rethinking Work in Industry 5.0: Leveraging Technology for an Ageing Workforce

**DOI:** 10.1002/puh2.70130

**Published:** 2025-09-23

**Authors:** Gaia Vitrano, Guido J. L. Micheli

**Affiliations:** ^1^ Department of Management Economics and Industrial Engineering Politecnico Di Milano Milan Italy

**Keywords:** ageing workforce, human–machine collaboration, inclusive workplace, Industry 5.0, technology integration

## Abstract

The workforce in many industries is ageing. With longer life expectancy and extended retirement ages, the proportion of older workers is growing, leading to challenges such as reduced physical and cognitive abilities. However, older workers bring valuable experience, knowledge, and skills that can benefit organizations. Technologies have a crucial role in this challenge, that is, in reducing physical and cognitive loads and improving workplace safety. Within the Industry 5.0 paradigm, the focus is moving away from solely automation and productivity toward improving human abilities and promoting worker well‐being. Industry 5.0 aims to create more personalized, efficient, and flexible work environments that can adapt to diverse worker needs, including those of older workers. This study aims to investigate the challenges and opportunities associated with an ageing workforce and how a human–machine collaboration can enhance their productivity and well‐being, promoting a human‐centric approach that leverages their strengths while addressing their evolving needs.

## 1 Introduction

Today, ageing represents one of the most significant concerns that humanity has to face. Due to the concurrent effects of declining birth rates and increasing longevity, the number and proportion of elderly individuals are rising worldwide, leading to considerable repercussions for economic growth, employment, consumption, and fiscal balance. According to the World Health Organization (WHO), the number of people aged 60 years and older was 1 billion in 2019, with projections estimating an increase to 1.4 billion by 2030 and 2.1 billion by 2050 [1]. This trend is expected to accelerate, particularly in developing countries. Therefore, this historical shift in global population distribution toward older ages requires significant structural adjustments across all sectors of society [2].

The work environment is consequently undergoing the same transformation due to a general change in the workforce characteristics. The participation rate of workers aged 50–64 in the European Union countries has increased from 36% in 2000 to 63% in 2020, as reported by the Organization for Economic Cooperation and Development [3]. Furthermore, many governments are implementing policies to raise the retirement age to ensure the sustainability of pension systems, resulting in a growing number of individuals working beyond the traditional retirement threshold.

As labor force participation among older workers increases, concerns arise regarding their long‐term employability and productivity. Ageing affects both physical and cognitive capacities, influencing performance in roles that require manual dexterity and mental agility, such as assembly or manufacturing tasks [4, 5].

Two major implications emerge: (1) an increase in exposure to hazards and in the risks of developing occupational diseases and physical problems; (2) burdens concerning the work ability of those aged over 65. These issues are particularly pressing in physically demanding jobs, where ageing‐related limitations can significantly impact productivity and safety. However, it is essential to recognize that ageing also brings advantages, including accumulated experience, expertise, strategic thinking, and problem‐solving skills, which can enhance productivity. Addressing these challenges is essential to ensuring sustainable workforce participation and preserving the functional abilities, both physical and cognitive, of older employees.

In the following sections, this work examines the implications of an ageing workforce (AW), delving into both its strengths and weaknesses, and analyzes its role within the framework of the human‐centric approach of Industry 5.0. It further discusses targeted interventions to support sustainable workforce participation and finally presents future directions for research.

## 2 AW and Technology

This article presents a review of current evidence on the evolving needs of an AW in technologically advanced workplaces. To provide a broad and reliable overview, we conducted a structured literature search across major academic databases, including Scopus and Web of Science, and policy reports relevant to ageing and technology in the workplace. The search strategy combined keywords (including variations in terminology and spelling) related to “AW/older workers,” “cognitive and physical abilities,” “musculoskeletal disorders (MSDs),” “mental workload,” “Industry 5.0 and human–machine collaboration,” “supportive technologies,” and “workplace interventions.”

**FIGURE 1:**
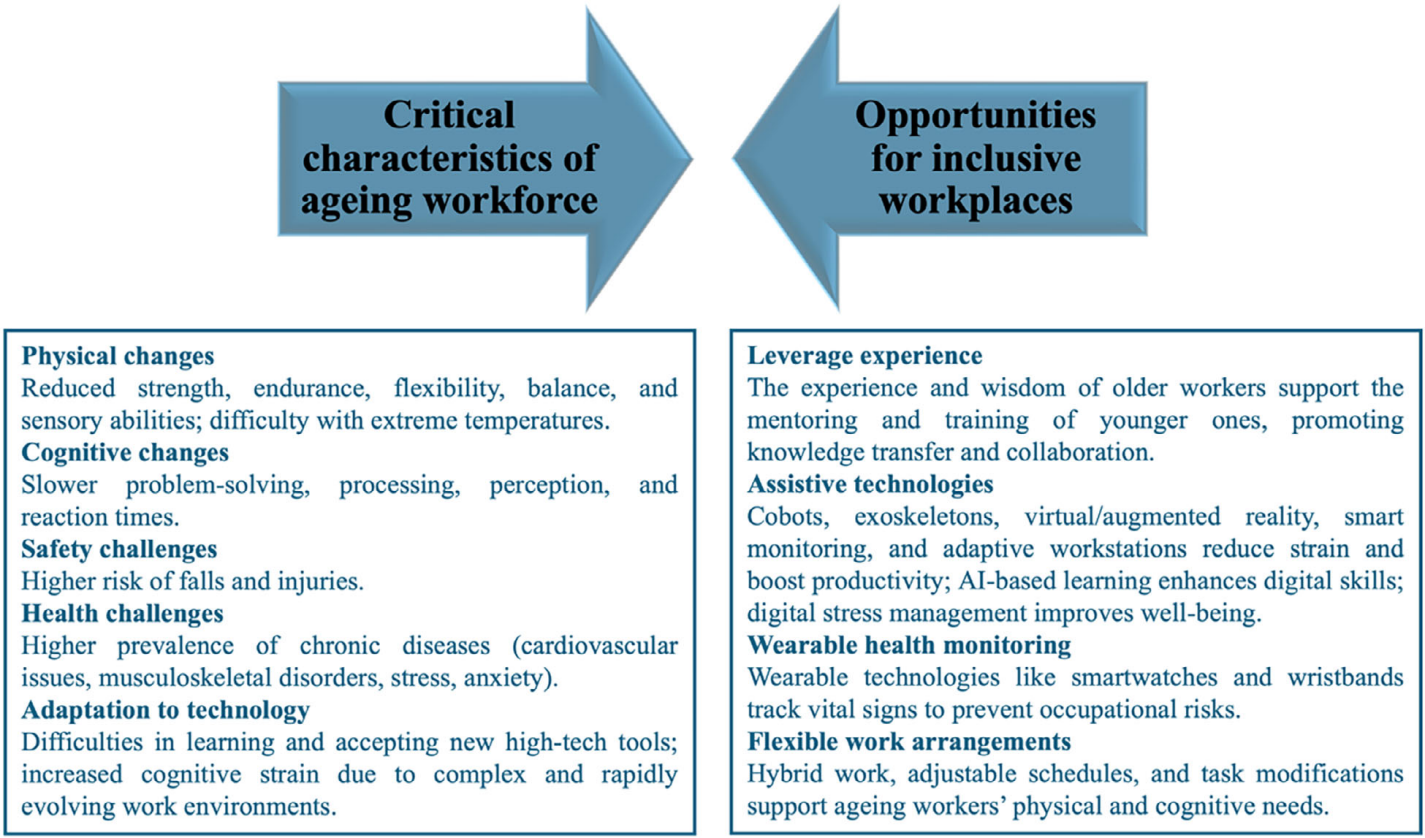
Key considerations for an AW—challenges and opportunities for inclusive workplaces.

The first part of this section provides an overview of existing research on AW dynamics, including physical and cognitive abilities. The second part focuses on Industry 5.0, human–machine collaboration, and how these principles can be applied to an AW, leveraging automation, AI‐driven support systems, and ergonomic advancements to enhance employability and workplace inclusivity. The last part discusses interventions that can be developed in this context to generate adaptive work environments and technological assistance, improving workforce sustainability and reducing age‐related occupational risks. Figure 1 summarizes the key findings, highlighting both challenges and opportunities for creating inclusive workplaces.

### 2.1 AW Abilities

A report from EU‐OSHA [6] analyzes the changes that occur in ageing individuals within the workforce and examines the likely impacts of work along the life course on health and ability. As individuals age within the workforce, they experience a range of physical, cognitive, and health‐related changes that can significantly impact their abilities and overall work performance.

### 2.2 Physical Changes With Age

From the early twenties, individuals begin to experience a reduction in average aerobic power, with a decline of approximately 10% per decade, which makes it more challenging for older workers to maintain high levels of physical activity. Similarly, muscle strength and endurance decrease over time, with the rate of decline becoming more pronounced after the age of 65. These changes can affect an individual's ability to perform physically demanding tasks and may necessitate adjustments in job roles or work environments to adapt to their needs [7].

Both postural and functional balance decline with age, increasing the likelihood of falls and related injuries. Joint mobility also decreases, which can limit the range of motion and flexibility required for certain tasks. Hand dexterity, crucial for fine motor skills, diminishes as well, impacting tasks that require precision and coordination [2].

### 2.3 Cognitive Changes With Age

Cognitive abilities also change with age. Some specific cognitive functions, such as fluid intelligence, which involves problem‐solving and the ability to process new information, show a decline from as early as 45 years old [8]. There are two forms of intelligence: fluid and crystallized [8]. Fluid intelligence, which involves real‐time problem‐solving, tends to decline with age, whereas crystallized intelligence, which encompasses accumulated knowledge and verbal abilities, is better maintained and can even improve. Psychological changes, including weakened precision and slower perception speed, can impact task performance [5]. However, older workers often compensate for these declines by prioritizing accuracy over speed, resulting in a trade‐off that can still yield high‐quality work [4]. This means that older workers may excel in tasks that rely on experience and knowledge rather than rapid information processing. Mental characteristics, such as wisdom, language control, reasoning, and motivation to learn, can strengthen with age, contributing to an individual's ability to adapt and perform complex tasks. Key elements of cognitive performance, such as intelligence, knowledge, and language use, generally do not show significant decline until after the age of 70, suggesting that older workers can remain highly capable in many areas [9].

### 2.4 Age‐Related Changes in General Health

Chronic diseases, which are long‐lasting and progress slowly, become more common with age. Some of these diseases are work‐related and can significantly impact an individual's ability to perform their job [10]. According to EU‐OSHA [6], adverse working conditions, such as exposure to harmful substances or repetitive strain, can contribute to the development of health problems over time. Among the most significant cardiovascular changes in ageing individuals reduce the ability to respond to extreme temperatures, which can be particularly challenging in work environments with significant temperature variations. Hearing deterioration is also common, particularly in certain industries. Visual changes starting from the mid‐forties—including decreased clarity, impaired performance in low lighting, difficulties in judging distances and speeds, and challenges in distinguishing between certain colors—can affect an individual's ability to perform tasks that rely heavily on visual acuity.

The length of exposure to these conditions is crucial, as health effects can result from cumulative exposure or have long latency periods before manifesting, as seen with diseases related to asbestos exposure. Additionally, stress, depression, anxiety, headaches, and MSDs are more commonly reported among older workers [11].

### 2.5 AW and Industry 5.0

This scenario calls for innovative strategies to support older workers while maintaining their productivity and well‐being [12]. The literature emphasizes the importance of explicitly considering the age of operators in ergonomic and productivity‐related aspects [13]. Traditionally, machine design has focused on functionality, often neglecting the cognitive effects on human operators. However, technological evolution, particularly in Industry 5.0, has made human–machine interaction increasingly central [14]. Consequently, an anthropocentric approach that considers the cognitive and psychological conditions of the operator is necessary to ensure safety and productivity.

There is an increasing demand for technologies such as arm‐based robots, exoskeletons, smart working tools, and immersive virtual reality to manage “age‐friendly” production and logistics systems. These technologies have the potential to maintain the productivity, quality, and well‐being of the AW [12]. According to Ranasinghe et al. [15], it is crucial for empirical research to explore how organizations can effectively introduce and implement these new technologies by considering the diverse physical and cognitive abilities of older workers.

Current European regulations recognize the importance of assessing mental workload but do not provide practical tools for measurement [14]. Cognitive psychology and neuroergonomics offer scientific methods to address this issue, allowing for the identification of critical cognitive states that can lead to operational errors. The goal is to design systems that maintain the operator in a state of cognitive comfort, reducing the risk of errors and improving performance.

Technologies, such as virtual reality, augmented reality, collaborative robots (cobots), and exoskeletons, have demonstrated their ability to reduce fatigue, stress, and depression, providing relaxation and significant benefits for older individuals [4]. These technologies are particularly useful for training, ergonomic design, and simulating safe working environments. Additionally, wearable technologies like smartwatches and wristbands can continuously monitor physiological states, such as heart rate, rhythm, and skin temperature. This continuous monitoring is especially relevant for workers in severe environments and older workers [16].

Looking ahead, it is crucial to focus on age‐friendly assistive and collaborative technology solutions and their interaction with the AW [17]. Challenges may arise due to the difficulties older workers face in training and accepting the latest high‐tech devices [4, 15]. Although these technologies are designed to reduce physical demands on workers, they introduce new operational risks. These risks require operators to adapt to more sophisticated and flexible interaction modalities, blending cognitive soft skills not previously demanded in traditional manufacturing settings [18].

Measuring cognitive load and adapting systems based on the operator's physiological responses is crucial for improving work ability and safety at the workplace [14], shifting from a machine‐ to a human‐centric approach [16]. This approach is particularly relevant for supporting an AW, ensuring that they remain productive and engaged.

### 2.6 Interventions for an Integrated Workforce

As the workforce ages, organizations must adopt strategic interventions that ensure the safety, well‐being, and productivity of older employees while leveraging the benefits of advanced technologies [5]. Interventions, previously focused on traditional safety measures, must now be adapted to the dynamic interplay of human workers and collaborative technologies [18, 19].

Ergonomics remains a fundamental aspect of workplace interventions, particularly for an AW. Industry 5.0 emphasizes human–machine collaboration, necessitating the development of adaptive workstations and intelligent ergonomic solutions, such as exoskeletons, AI‐driven workplace monitoring, and customized workstation adjustments [20].

For ageing workers, the risk of chronic diseases increases, and wearable health monitoring devices, AI‐driven health analytics, and personalized health plans can help track and mitigate risks related to high blood pressure, cardiovascular diseases, and mental health challenges [16]. Additionally, stress management programs supported by digital solutions can enhance well‐being and productivity [4].

Furthermore, the rapid technological advancements in Industry 5.0 require continuous learning, especially for older employees who may face digital skill gaps [21]. Implementing age‐inclusive training programs that leverage AI‐based adaptive learning systems and training with collaborative robots (cobots) can facilitate skill acquisition, reducing resistance to technological changes and enhancing workers’ confidence and ability to adapt to new work processes [18].

## 3 Discussion and Future Steps

On the basis of previous evidence, future research on the AW should focus on several key areas to ensure that technological advancements align with their needs while leveraging their strengths. Interventions for the prevention of health and safety must evolve to meet the unique challenges and opportunities presented by the AW. By integrating ergonomics, disease prevention, and targeted training within a technologically advanced setting, organizations can enhance workplace safety, improve employee well‐being, and maintain productivity. These interventions must be context‐specific, leveraging Industry 5.0 innovations while ensuring a human‐centric approach that values the experience and capabilities of ageing workers. Key directions for future research are included below.

### 3.1 Directions for Further Development

Understanding physical and cognitive changes in ageing workers

- To better understand how ageing affects workers, it is essential to monitor the progression of age‐related changes in both physical and cognitive abilities—for example, decline in aerobic capacity, muscle strength, joint mobility, and sensory abilities—which will support the development of specific guidelines addressing the negative aspects and leveraging their strengths. By doing so, we can ensure that the experience and accumulated knowledge of older workers are effectively utilized, allowing them to continue contributing meaningfully to their roles.

Leveraging the strengths of an AW

- Organizations might utilize older workers as mentors and trainers for younger employees, fostering knowledge transfer and intergenerational collaboration. Job roles should be designed to emphasize the experience and wisdom of older workers. Moreover, flexible work arrangements and adaptive job designs should be encouraged to maximize productivity and, at the same time, minimize physical strain, creating an environment where older workers can continue to be active and contribute effectively.

Enhancing human–machine collaboration for an age‐inclusive workforce

- Assessing the impact of Industry 5.0 innovations on workforce inclusivity is essential. It is important to ensure that health and safety interventions do not inadvertently exclude older workers, creating a more supportive and adaptable work environment for all employees. Technologies, such as AI‐driven support systems, exoskeletons, and collaborative robots (cobots), should be tailored to support older workers, ensuring they remain productive and engaged. To bridge skill gaps in an age‐inclusive workforce, it is important to develop adaptive learning platforms that leverage AI to provide personalized training. Immersive training environments and mentorship programs can foster technology adoption among older workers.

Considering the long‐term impact of assistive technologies

- Another side of assistive technologies to be investigated is the long‐term effects of assistive technologies like wearable monitoring devices and virtual/augmented reality applications. Studying the potential risks associated with prolonged human–machine interaction, such as cognitive fatigue, stress, and ergonomic strain, and proposing effective mitigation strategies, will further support the development of age‐friendly workplaces. Therefore, investigating the feasibility of using real‐time physical and cognitive monitoring to adjust task demands becomes essential to analyze and prevent workplace fatigue, stress, and injuries.

Discussing policies and regulations for an age‐inclusive workforce

- To better protect ageing workers in working environments, it is essential to assess existing labor laws and occupational health regulations. Refining current regulations will help to further develop industry standards and policy guidelines for implementing age‐friendly technological solutions that promote inclusivity and productivity. In this matter, policymakers and industry leaders should work together to embed AW considerations into future workplace transformations.

By prioritizing these research areas, organizations and policymakers can create a sustainable, inclusive, and technologically advanced work environment that supports the needs of an AW. A human‐centric approach to Industry 5.0 would enhance workplace productivity and keep experienced workers engaged.

## Author Contributions

**Gaia Vitrano:** conceptualization, investigation, writing – original draft, writing – review and editing, visualization, validation, supervision. **Guido J. L. Micheli:** validation, writing – original draft, investigation, conceptualization.

## Ethics Statement

This short communication is based solely on the analysis of existing literature and does not involve any studies with human participants or animals.

## Conflicts of Interest

The authors declare no conflicts of interest.

## Data Availability

The data that support the findings of this study are available from the corresponding author upon reasonable request.
